# Dependence of energy balance and hypothalamic neuropeptide gene expression on initial tumor load in mice

**DOI:** 10.3389/fonc.2026.1783555

**Published:** 2026-05-19

**Authors:** Maria Licursi, Caleb Morden, Deogratias G. Riwa, Haley Briggs, Kensuke Hirasawa, Michiru Hirasawa

**Affiliations:** Division of Biomedical Sciences, Faculty of Medicine, Memorial University, St. John’s, NL, Canada

**Keywords:** cancer anorexia, cancer cachexia, cancer mouse model, hypocretin, hypothalamus, melanin-concentrating hormone, melanocortin, neuroinflammation

## Abstract

**Introduction:**

Animal models of cancer anorexia/cachexia are instrumental in investigating the underlying mechanisms. However, even among reports on the same cancer model, experimental procedures can vary, hindering the comparisons between studies.

**Methods:**

To determine the impact of the number of implanted cancer cells on experimental outcomes, we directly compared tumor-bearing (TB) mice inoculated with Lewis-lung carcinoma at 1x10^6^ cells (TB_high_) or 0.5x10^6^ cells (TB_low_).

**Results:**

While both TB groups showed lower weight gain and adiposity than vehicle controls, TB_low_ mice displayed a larger reduction, possibly due to prolonged time to the endpoint. Body weight gain was highly correlated with food efficiency in all groups, while correlation with food intake was found only in TB_high_ group. Using these models, hypothalamic energy balance-related mRNA expression was examined, which found that *Pomc* and *Hcrt* were downregulated in TB_low_ mice, whereas *Agrp* was upregulated in TB_high_ mice. *Npy* and *Pmch* were unaltered. Expression of respective neuropeptide receptor mRNA, *Mc3r*, *Mc4r*, *Hcrtr2*, and *Mchr1*, were also not altered. Furthermore, hypothalamic expression of inflammatory genes *Ccl2* and *Il6* was tightly correlated with each other in all groups, while *Ccl2* was negatively correlated with *Hcrt* and *Pomc* in TB_low_ mice, suggesting a role for CCL2 in regulating neuropeptide expression.

**Discussion:**

The metabolic profile and hypothalamic gene expression can vary with differential initial tumor load, suggesting that the relationship between systemic energy balance and cancer progression is complex and not strictly linear. This finding should be carefully considered in the design and interpretation of future animal experiments and clinical studies.

## 1 Introduction

Many cancer patients present with a decrease in appetite, body weight, adipose and muscle mass associated with reduced physical functioning, tolerance to anticancer therapy, and quality of life (1). Anorexia and cachexia are estimated to occur in up to 80% of individuals with advanced cancers and causes over 20% of cancer-related deaths (2, 3). Given the prevalence and serious consequences, it is important to understand the underlying mechanisms and identify new therapeutic targets for cancer-related anorexia and cachexia.

Body weight is maintained by the coordinated regulation of energy intake and expenditure by the brain. This central energy balance circuitry resides within the hypothalamus, which regulates physiological processes and behavior according to metabolic demand (4). Orexigenic agouti-related peptide/neuropeptide Y (AgRP/NPY) neurons and anorexigenic proopiomelanocortin (POMC) neurons in the arcuate neurons integrate nutrient and adiposity signals from periphery and orchestrate so-called second-order effector neurons to regulate energy homeostasis (5). Among the second-order neurons are two distinct cell populations distributed within the lateral, perifornical, and dorsomedial hypothalamus, namely melanin-concentrating hormone (MCH) neurons and hypocretin (orexin) neurons (6). MCH neurons promote weight gain by increasing food intake while suppressing energy expenditure, whereas hypocretin neurons promote wakefulness and food seeking with coordinated upregulation of metabolic rate and physical activity (7, 8). Together, these peptidergic neurons control different aspects of food intake and energy expenditure. Maladaptation of this energy balance circuitry is thought to result in cancer-related anorexia, such as the activation of anorexic melanocortin and serotonin signaling, and the hypothalamic-pituitary-adrenal axis (9, 10). Furthermore, changes in hypocretin neurons with implications for cancer-related fatigue and insomnia have been reported, although these reports suggest increased or decreased activity of the hypocretin system (11, 12), which may be due to differences in animal models. MCH neurons have been speculated to play a role in cancer-related metabolic and sleep disturbances (13), however, how these cells respond to the presence of tumor remains unclear.

There are many tumor-bearing (TB) animal models with varied levels of anorexia and cachexia (14). One such model is the Lewis-lung carcinoma (LLC) TB mouse model, which presents with anorexia (15, 16) and cachexia (15–24). However, in studies utilizing this model, the number of inoculated LLC cells ranged from 5 x 10^1^ to 5 x10^6^ cells (15–24) resulting in highly variable severity and time course of anorexia and/or cachexia, and time to endpoint. These issues raise the need to optimize the TB mouse model, which is one of the most common animal models for studying cancer-related anorexia and cachexia.

Here, we sought to determine how the initial tumor load impacts energy balance in mice. To test this, we made a direct comparison of TB mice inoculated with two different numbers of tumor cells. Our study indicates that physiological outcomes and the expression of energy balance-related hypothalamic genes depend on the initial tumor load, providing new insights into the role of hypothalamic neuropeptides in cancer anorexia and cachexia.

## 2 Materials and methods

### 2.1 Animals

All experiments were carried out in accordance with the Canadian Council on Animal Care (CCAC) guidelines and as approved by Memorial University Institutional Animal Care Committee (Protocol 18-01-MH). Male C57BL/6NCrl mice (8–9 weeks old) were purchased from Charles River Laboratories, QC, Canada (RRID: IMSR_CRL:027). Upon arrival, mice were singly housed and allowed to acclimatize for a week within the animal care facility at the Health Sciences Centre under 12h light/12h dark cycle (lights on at 8:00AM- 8:00PM), and had access to standard rodent chow (Prolab RMH 3000) and water *ad libitum*. Body weight and food intake were measured 3 times a week throughout the experimental period. Food efficiency was calculated as the ratio of total change in body weight and total food consumed.

### 2.2 Lewis lung carcinoma model

Mouse LLC cells (American Type Culture Collection, Manassas, VA, USA, RRID: CVCL_0391) were cultured in high-glucose Dulbecco’s modified Eagle’s medium (Corning, MA, USA) with 10% fetal bovine serum (HyClone, Cytiva, USA origin), 1 mM sodium pyruvate and antibiotic-antimycotic (Thermo Scientific). On the day of injection, cells were harvested, washed and counted, and cell number was adjusted in 100 μL of phosphate-buffered saline (PBS).

**Figure 1:**
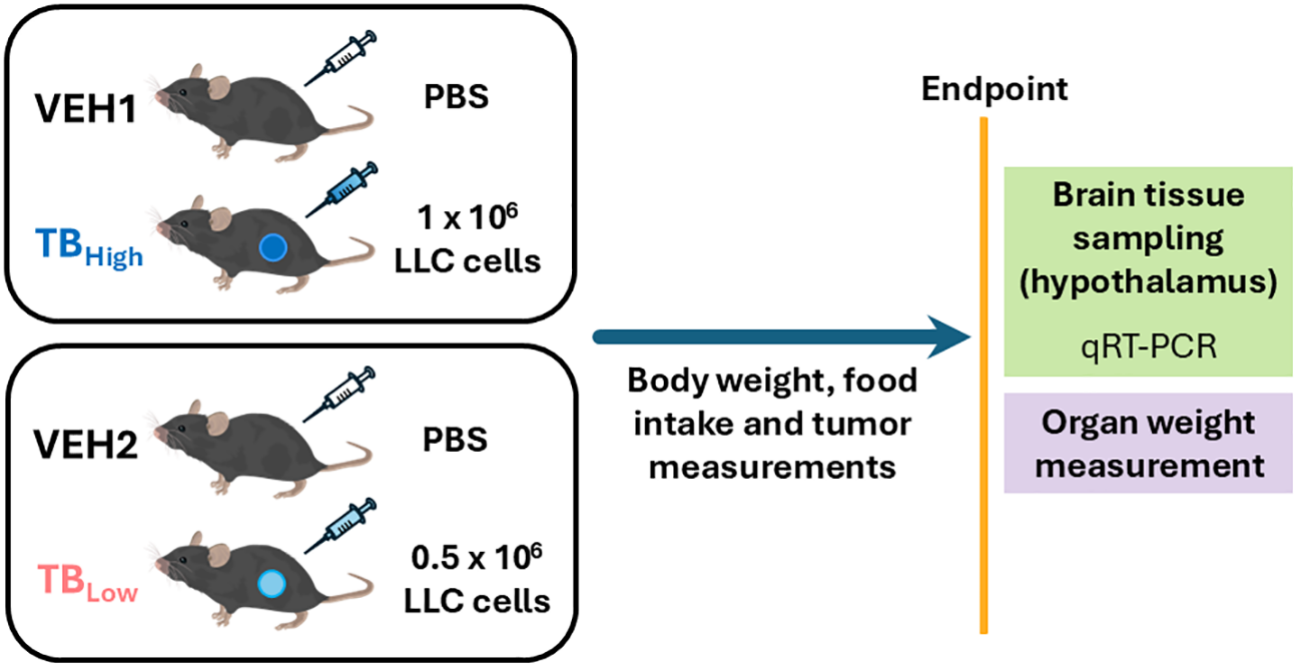
Experimental design.

Following acclimatization, mice were assigned to one of the three groups so that the group mean of the initial body weight was similar but otherwise randomly. Each group received a subcutaneous injection on the hind flank: (1) Vehicle (PBS 100 μL, n = 22), (2) TB_high_ (1 x 10^6^ LLC cells/100 μL PBS, n = 14) or (3) TB_low_ (0.5 x 10^6^ LLC cells/100 μL PBS, n = 10). The sample size was based on past studies on LLC-TB mice, which ranged from 4 to 16 mice/experimental group (15–24). Each cohort consisted of a vehicle group and one of the two TB groups (**Figure 1**). Data collection was not blinded as the number of implanted cells were noted on each animal ID card posted on their cage for welfare assessment.

Experimental endpoint was defined as the time when the criteria for euthanasia (humane endpoint) were met for multiple TB mice in a cohort. Specifically, we followed the CACC guidelines on humane endpoints for cancer research, defined as the tumor reaching a diameter of 17 mm on the long axis, the presence of an ulceration or infection, or body weight loss of >20%. All mice in a cohort were sacrificed for tissue sampling at the same experimental endpoint for consistency in elapsed time since implantation of cancer cells. Vehicle controls were also sacrificed at time points matching the corresponding TB groups and are designated as VEH_1_ (matched to TB_high_) and VEH_2_ (matched to TB_low_).

### 2.3 Tissue sampling

Organ sampling was performed between 12:00PM and 5:00PM with the order of sacrifice randomized between groups. Mice were anaesthetized with inhalation of isoflurane (4%) and decapitated. The brain was quickly removed and a coronal slice (1.25 mm thick) containing the hypothalamus was generated using a vibratome (VT-1000, Leica Microsystems) in ice cold artificial cerebrospinal fluid composed of 126 mM NaCl, 2.5 mM KCl, 1.2 mM NaH_2_PO_4_, 2 mM CaCl_2_, 1.2 mM MgCl_2_, 18 mM NaHCO_3_, 2.5 mM glucose, continuously bubbled with 95% O_2/_5% CO_2_. Then, the whole hypothalamus was isolated and stored at -80 °C until further use. Organ weights were measured immediately following sacrifice. Corrected body weight change was calculated as the difference between the body weight on the day of LLC cell injection and sacrifice with the tumor mass subtracted for TB groups.

### 2.4 Gene expression analysis

RNA was isolated from brain samples using TRIzol (Thermo Fisher) following manufacturer’s instructions. cDNA was synthesized from the RNA using the RevertAid H Minus First Strand cDNA Synthesis Kit (Thermo Scientific). Real-time quantitative polymerase chain reaction (qRT-PCR) was performed in triplicate on StepOnePlus (Applied Biosystems) using Power SYBR Green PCR Master Mix (Applied Biosystems). Primers used for this experiment are listed in **Table 1** (Integrated DNA Technology). Cycling conditions consisted of 95 °C for 10 minutes, 40 cycles at 95 °C for 15 seconds, 60 °C for 1 minute, followed by melt-curve analysis. The absence of nonspecific amplification was confirmed by observing a single peak in the melt-curve and the absence of amplification in the no-template control wells. For data analysis, the delta cycle threshold value of target genes were first normalized to respective GAPDH for each sample. Then, fold change relative to controls was calculated by normalizing to the average of respective age-matched VEH groups.

**Table 1:** qRT-PCR primers list.

Target mRNA	Forward primer sequence	Reverse primer sequence
*Pmch*	GCAGAAAGATCCGTTGTCGC	GTTTGGAGCCTGTGTTCTTTGA
*Hcrt*	GGCCTCAGACTTCTTGGGTATT	AGGGAACCTTTGTAGAAGGAAAGT
*Pomc*	CACGTGGAAGATGCCGAGAT	CCAGCGAGAGGTCGAGTTTG
*Agrp*	TGTGTAAGGCTGCACGAGTC	CATCCATTGGCTAGGTGCGA
*Npy*	CAGAACAAGGCTTGAAGA	GCAGACTGGTTTCAGGGGAT
*Mchr1*	CTCCTGTGTGGCTCTATGCC	TTCACGTACGCAGCAGTGAT
*Hcrtr2*	CACGGACTATGACGACGAGG	AGAGCCACAACGAACACGAT
*Mc3r*	GCTACACGGCCCATTTCAAC	AGCCGCAGAGAATCTCCTTG
*Mc4r*	GGGTCGGAAACCATCGTCAT	GCGAGCAAGGAGCTACAGAT
*Ccl2*	AACTGCATCTGCCCTAAGGT	AGGCATCACAGTCCGAGTCA
*Il6*	GAGACTTCCATCCAGTTGCCT	TGGGAGTGGTATCCTCTGTGA
*Gapdh*	ATGTGTCCGTCGTGGATCTGA	TGCCTGCTTCACCACCTTCTT

### 2.5 Statistical analysis

Data are presented as mean ± SEM. Statistical tests were performed using Prism 8 (Graphpad) and p<0.05 was considered significant. To compare between groups over time, two-way repeated measures (RM) ANOVA with Sidak’s multiple comparison test was used. For comparisons of 3 groups with similar standard deviation, one-way ANOVA with Tukey’s multiple comparisons test was performed. If Bartlett’s test found significantly different standard deviation among the groups, Kruskal-Wallis test followed by Dunn’s multiple comparisons test was used instead. ANOVA and Kruskal-Wallis test results on the main effect (p values) are indicated in the Results whereas significant results of multiple comparisons test are indicated with asterisks in Figures (*p<0.05, **p<0.01, ***p<0.001, ****p<0.0001). Non-significant results in multiple comparisons test (p≥0.05) are not labeled. Least squares linear regression analysis was performed to assess correlation between two factors.

## 3 Results

### 3.1 Characteristics of LLC tumour-bearing mice

To characterize LLC-TB mice, we compared body weight and food intake of mice injected with different number of LLC cells to those treated with vehicle only. We chose to test 1 x 10^6^ (TB_high_) and 0.5 x 10^6^ (TB_low_) LLC cells as these cell numbers are the most commonly utilized and have been shown to induce anorexia and/or cachexia in mice (16, 18, 19, 22). In the present study, TB_high_ and TB_low_ groups reached the experimental endpoint at a time range of 15–16 days and 20–29 days post-injection, respectively.

**Figure 2:**
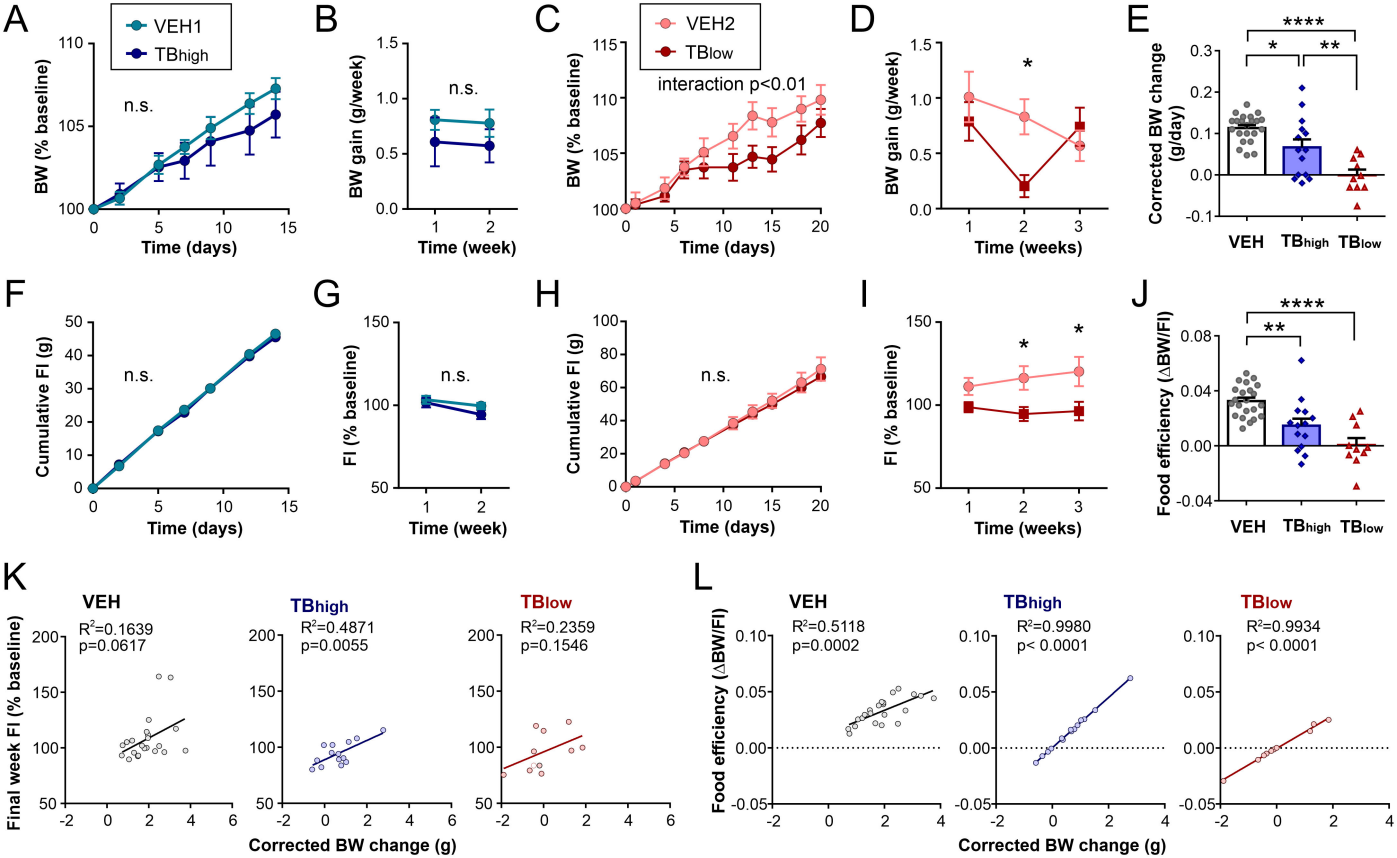
Physiological characteristics of LLC tumor-bearing (TB) mice depend on the initial tumor load. **(A, B)** Body weight (BW) **(A)** and weekly BW gain **(B)** of TB_high_ mice (injected with 1x10^6^ LLC cells) and time-matched vehicle control (VEH1). **(C, D)** BW **(C)** and weekly BW gain **(D)** of TB_low_ mice (injected with 0.5x10^6^ LLC cells) and time-matched vehicle control (VEH2). For **(A–D)**, body weight is not corrected for tumor weight. Day 0 represents the time of LLC cell injection. **(E)** BW change post-injection corrected for tumor weight, normalized by the number of days to sacrifice. **(F–I)** Cumulative **(F, H)** and weekly **(G, I)** food intake (FI) post-injection for TB_high_ **(F, G)** and TB_low_ mice **(H, I)**. **(J)** Overall food efficiency post-injection, calculated as a ratio of total BW gained (corrected for tumor weight) and total food consumed. **(K, L)** Relationship between corrected BW change and FI during the final week **(K)** or food efficiency **(L)**. Mean ± SEM are shown. Scatter plots depict individual mice. VEH is a combined group of VEH1 and VEH2. Significant results (p<0.05) are indicated. *p<0.05, **p<0.01, ****p<0.0001. n.s.: no significant main effect.

The time course of body weight changes and food intake in the TB groups were compared to their respective age-matched vehicle controls (VEH_1_ and VEH_2_). During the post-injection period, TB_high_ mice showed a similar rate of body weight gain as vehicle controls (VEH_1_ n=13 vs. TB_high_ n=14, two-way RM ANOVA, body weight: treatment p=0.5552, time p<0.0001, interaction p=0.5681, **Figure 2A**; weekly body weight gain: treatment p=0.1953, time p=0.8359, interaction p=0.9864, **Figure 2B**). On the other hand, TB_low_ mice showed significantly lower weight gain than controls during the second week post-injection, which rebounded in the third week (VEH_2_ n=9 vs. TB_low_ n=10, two-way RM ANOVA, body weight: treatment p=0.1637, time p<0.0001, interaction p=0.0065, **Figure 2C**; weekly body weight gain: treatment p=0.0995, time p=0.0872, interaction p=0.0752, **Figure 2D**).

While VEH_2_ gained more weight than VEH_1_ post-injection due to the one-week difference in the timing of sacrifice, these two vehicle groups did not significantly differ in weekly weight gain, food efficiency, and the majority of organ weights (**Supplementary Figure 1**). As they underwent the same treatment (vehicle injection) and displayed the same energy balance profile, they were combined as one control group (VEH) for the endpoint comparisons. Correcting body weight for tumor mass (final body weight - tumor weight) revealed that both TB groups gained significantly less weight than their vehicle-injected counterparts, with TB_low_ displaying more severe reduction in the daily rate of weight gain than TB_high_ (one-way ANOVA, p<0.0001, **Figure 2E**). These results indicate that the rebound weight gain in TB_low_ mice seen in the third week is likely due to tumor growth.

Meanwhile, neither cumulative nor weekly food intake post-injection was significantly different between TB_high_ and control mice (two-way RM ANOVA, cumulative food intake: treatment p=0.8221, time p<0.0001, interaction p=0.6637, **Figure 2F**; weekly food intake: treatment p=0.3033, time p=0.0016, interaction p=0.2786, **Figure 2G**). Cumulative food intake of TB_low_ mice was also not different from controls; however, food consumption was significantly less during the second and third week (two-way RM ANOVA, cumulative food intake: treatment p=0.6859, time p<0.0001, interaction p=0.8924, **Figure 2H**; weekly food intake: treatment p=0.0181, time p=0.5339, interaction p=0.1802, **Figure 2I**). Food efficiency, an indirect measure of energy expenditure, was also decreased by the presence of tumor, particularly in TB_low_ mice (one-way ANOVA p<0.0001, **Figure 2J**).

We next assessed how the change in body weight corresponded with other energy balance measures in these mice. We found that corrected body weight change was significantly correlated with food intake during the final week in TB_high_ but not in TB_low_ mice or vehicle controls (linear regression, VEH: n=22, p=0.0617; TB_high_: n=14, p=0.0055, TB_low_: n=10, p=0.1546, **Figure 2K**). Furthermore, corrected body weight change was strongly correlated with food efficiency in all groups (VEH: p=0.0002; TB_high_: p<0.0001, TB_low_: p<0.0001, **Figure 2L**). These results suggest that the main contributor to lower body weight gain in TB mice is diminished food efficiency, with some contribution from reduced caloric intake.

**Figure 3:**
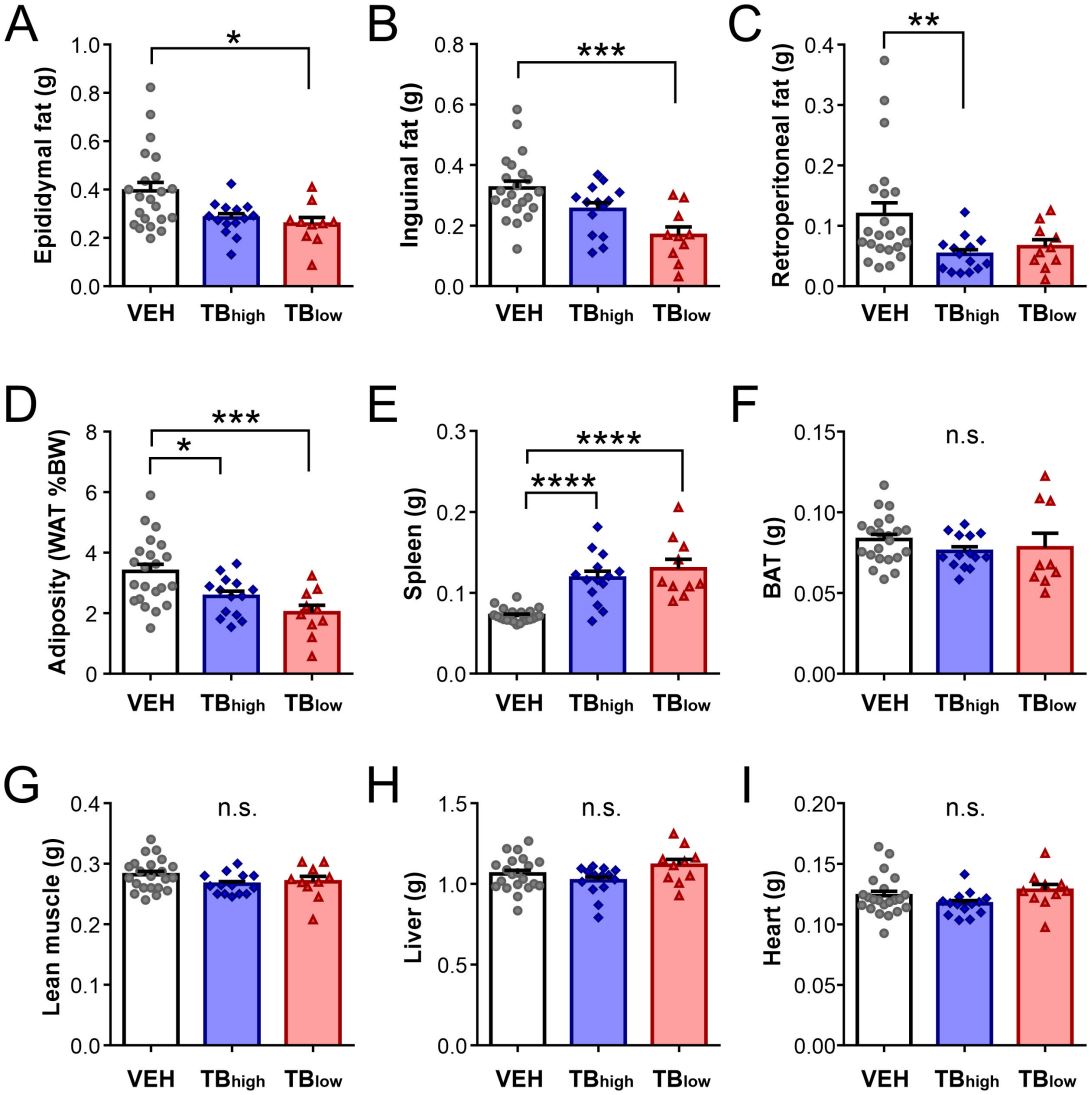
LLC tumor-bearing mice display adipose wasting. **(A–C)** White adipose tissue (WAT) mass, namely epididymal **(A)**, inguinal **(B)**, and retroperitoneal fat **(C)**. **(D)** Adiposity represented as the sum of WAT mass (epididymal, inguinal and retroperitoneal) normalized to BW. E-I) Weight of the spleen **(E)**, brown adipose tissue (BAT) **(F)**, lean muscles including the gastrocnemius and soleus muscles **(G)**, liver **(H)**, and heart **(I)**. Mean ± SEM are shown. *p<0.05, **p<0.01, ***p<0.001, ****p<0.0001. n.s.: no significant main effect.

At the time of sacrifice, the mass of various organs was assessed. One or more white adipose tissue (WAT) such as epididymal, inguinal and retroperitoneal fat pads were reduced in TB groups relative to controls (VEH n=22 vs. TB_high_ n=14, TB_low_ n=10; epididymal fat: Kruskal-Wallis test p=0.0195, inguinal fat: one-way ANOVA p=0.0004; retroperitoneal fat: Kruskal-Wallis test p=0.0094, **Figures 3A–C**). Overall, TB groups had significantly less adiposity (one-way ANOVA p=0.0008, **Figure 3D**). Spleen was enlarged in both TB groups (Kruskal-Wallis test p<0.0001, **Figure 3E**). In contrast, no differences were found in brown adipose tissue (BAT: Kruskal-Wallis test p=0.2470, **Figure 3F**), lean muscle (sum of gastrocnemius and soleus muscles: one-way ANOVA p=0.1474, **Figure 3G**), liver (one-way ANOVA p=0.0924, **Figure 3H**), and heart mass (one-way ANOVA p=0.1793, **Figure 3I**).

Altogether, our study shows that TB mice display less food intake, body weight and adiposity. However, the degree of reduction depends on the initial tumor load, with inoculation of a lower number of cancer cells resulting in a more robust metabolic phenotype. This difference appears to be due to dynamic imbalance between food intake and energy expenditure.

### 3.2 Hypothalamic gene expression in LLC Tumor-bearing mice

**Figure 4:**
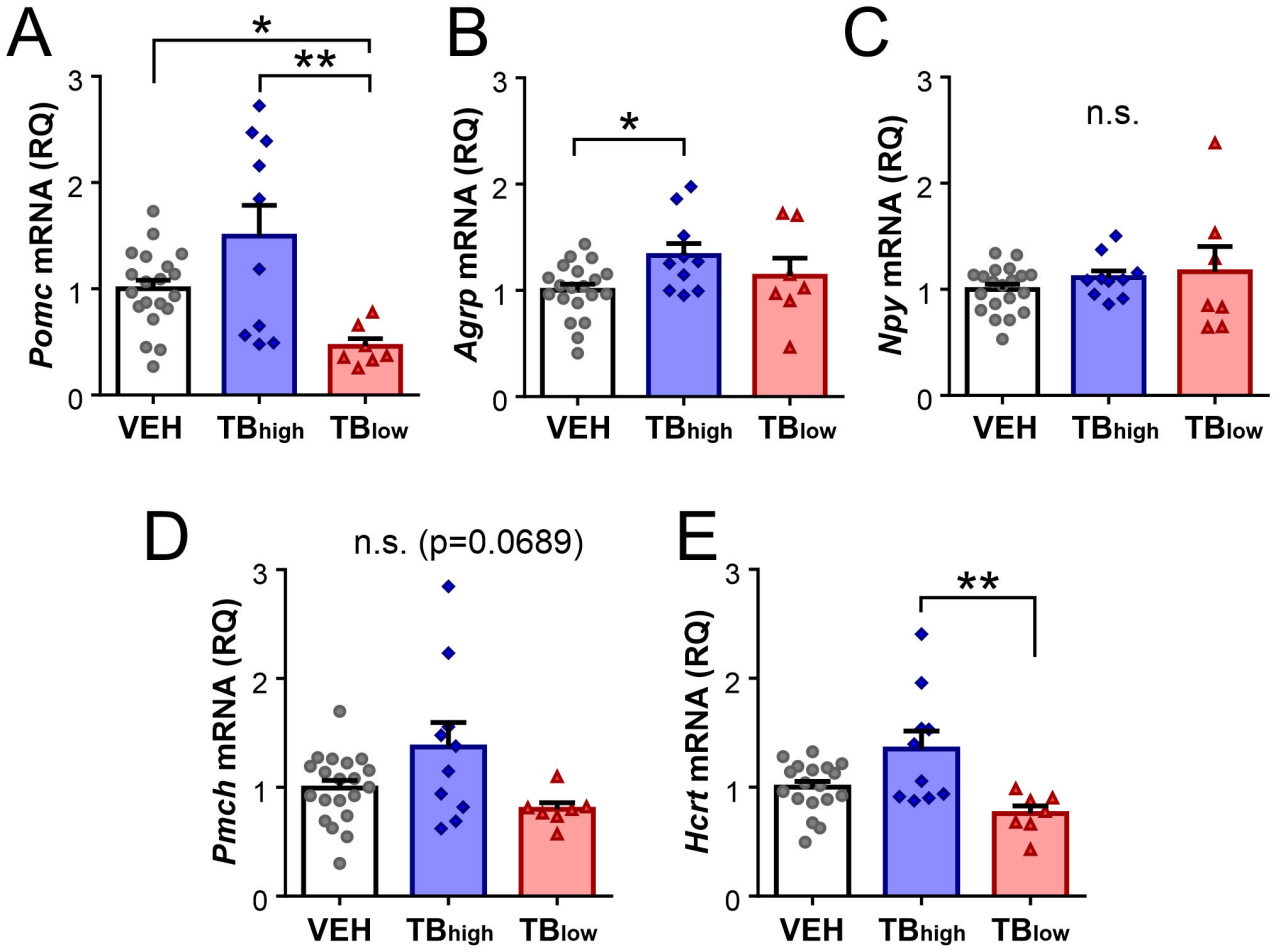
Changes in expression of energy balance-related hypothalamic peptide genes depend on initial tumor load. Relative mRNA expression of *Pomc* **(A)**, *Agrp* **(B)**, *Npy* **(C)**, *Pmch* **(D)** and *Hcrt* **(E)** in TB_high_ and TB_low_ mice. Measured values were normalized to GAPDH, then normalized to respective age-matched vehicle controls. Symbols depict individual mice. Mean ± SEM are shown. *p<0.05, **p<0.01. n.s.: no significant main effect.

**Figure 5:**
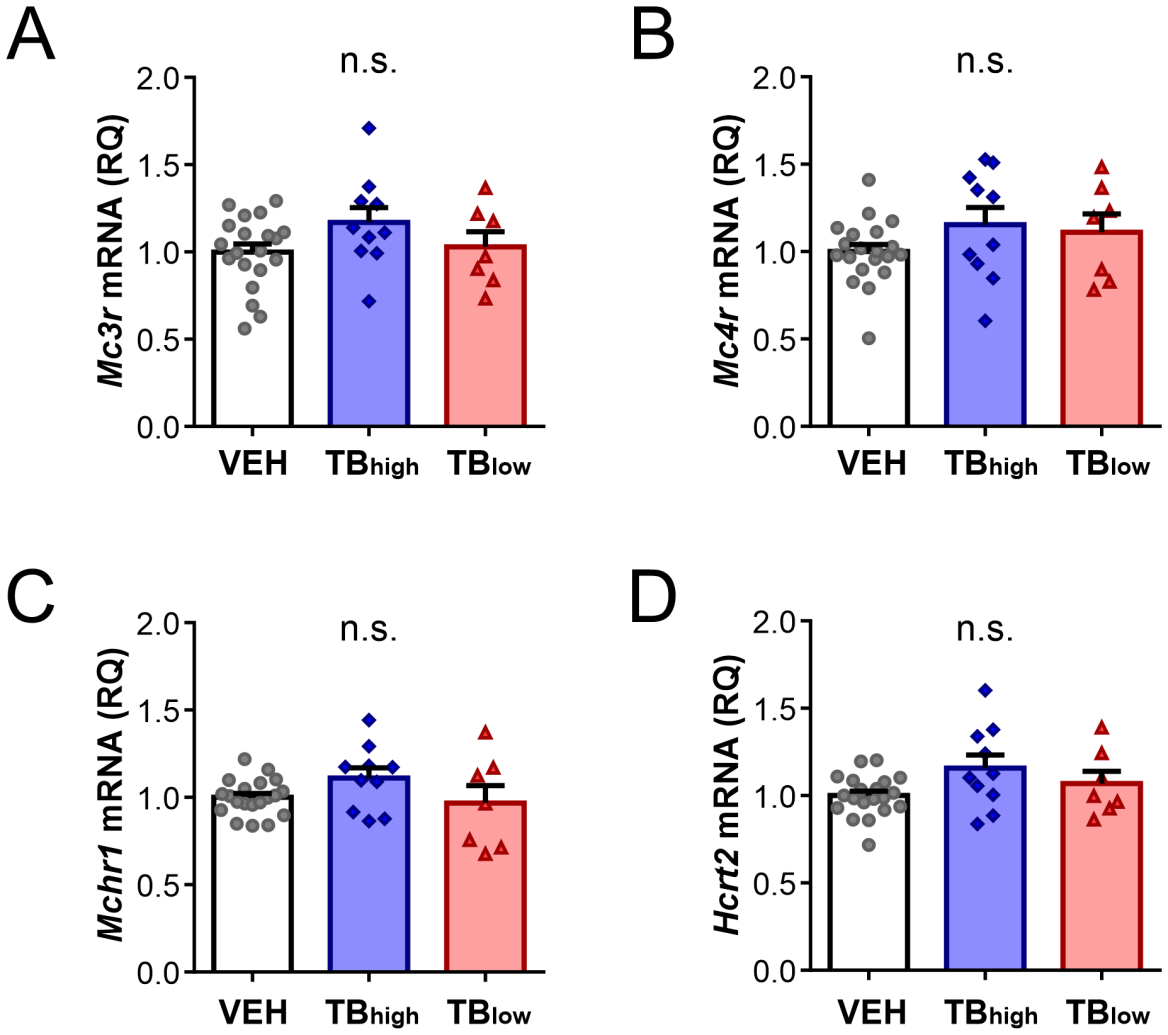
Expression of energy balance-related peptide receptor genes in the hypothalamus is not affected by the presence of tumor. Relative hypothalamic expression of melanocortin receptor *Mc3r* **(A)** and *Mc4r* **(B)**, MCH receptor *Mchr1* **(C)**, and hypocretin receptor *Hcrt2* **(D)** in vehicle control and TB mice. Measured values were normalized to GAPDH, then normalized to respective age-matched vehicle controls. Symbols depict individual mice. Mean ± SEM are shown. n.s.: no significant main effect.

The hypothalamus is positioned to integrate peripheral signals informing the presence of cancer and in turn adjust energy balance. To investigate whether the presence of tumor affects hypothalamic gene expression, RT-qPCR was performed on energy balance-related neuropeptides and their receptors. The analysis included the arcuate neuropeptide genes *Pomc* expressed by anorexigenic POMC neurons, and *Agrp* and *Npy* expressed by orexigenic AgRP/NPY neurons. These neurons act antagonistically to inhibit or promote body weight gain, respectively (5). *Pomc* level was significantly lower in TB_low_ group relative to controls or TB_high_ (VEH n=20, TB_high_ n=10, TB_low_ n=7, Kruskal-Wallis test p=0.0033, **Figure 4A**) whereas *Agrp* was higher in TB_high_ compared to controls but not TB_low_ (one-way ANOVA p=0.0460, **Figure 4B**). No change in *Npy* mRNA was found in TB mice (Kruskal-Wallist test p=0.6322, **Figure 4C**). Within the lateral hypothalamus, MCH and hypocretin neurons play complementary roles in regulating food intake, energy expenditure and sleep (7). In TB mice, *Pmch* levels showed a trend but not a significant difference (Kruskal-Wallis test p=0.0689, **Figure 4D**). *Hcrt* mRNA expression was significantly lower in TB_low_ group compared to TB_high_ (Kruskal-Wallis test p=0.0071; **Figure 4E**). In contrast to neuropeptide genes, their receptor mRNA levels did not show significant difference among the groups (*Mc3r*: one-way ANOVA, p=0.1636; *Mc4r*: one-way ANOVA, p=0.2340; *Mchr1*: Kruskal-Wallis test, p=0.2006; *Hcrtr2*: Kruskal-Wallis test, p=0.1731. **Figures 5A–D**).

**Figure 6:**
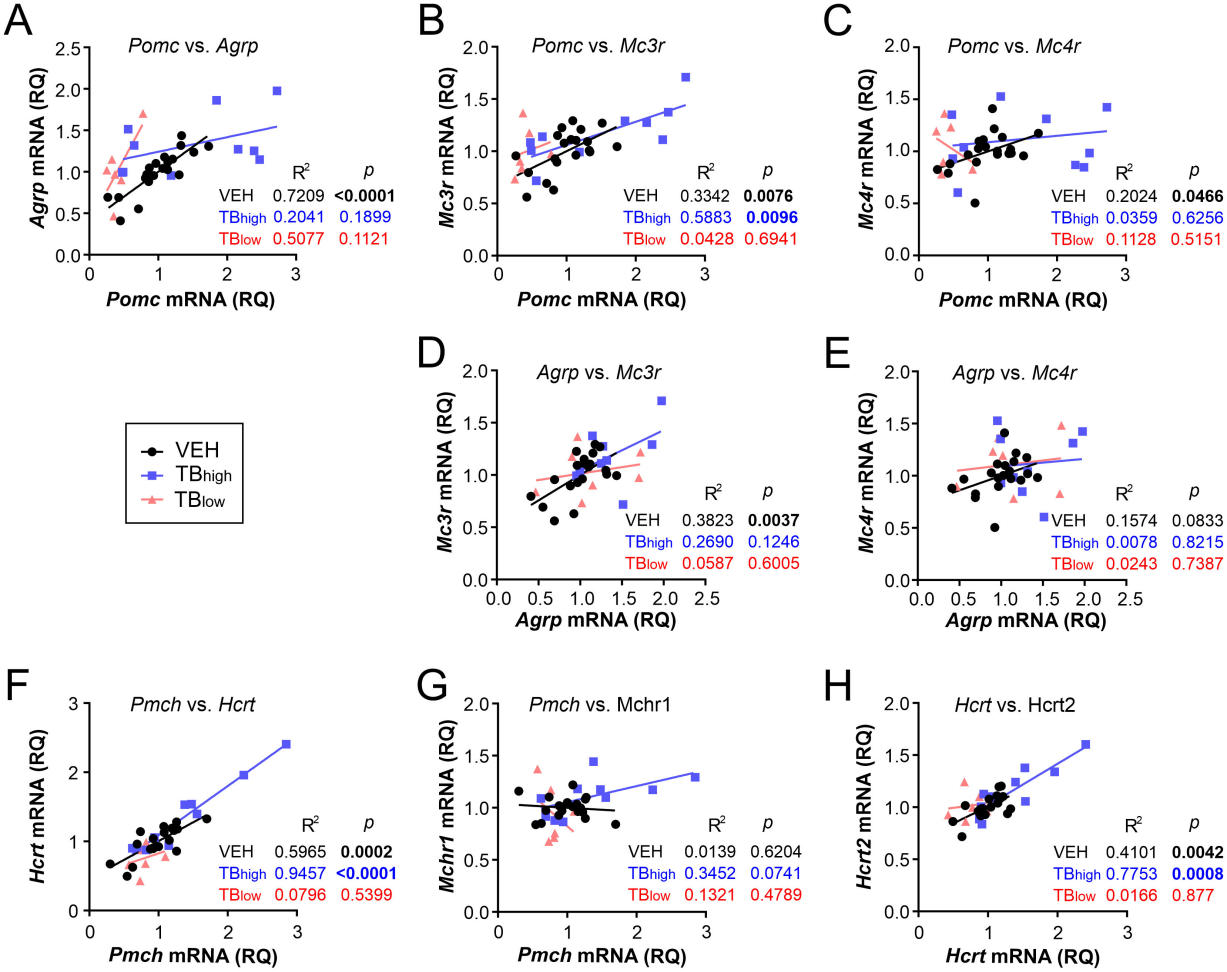
Relationship between peptide and receptor genes expression. **(A)** Arcuate genes *Pomc* and *Agrp* are not correlated in TB mice. **(B, C)** *Pomc* level is correlated with *Mc3r* in TB_high_ mice **(B)** but not with *Mc4r* **(C)**. **(D, E)** *Agrp* mRNA level does not correlate with *Mc3r* or *Mc4r* in TB mice. **(F)** Lateral hypothalamic genes *Pmch* and *Hcrt* are highly correlated in TB_high_ mice. **(G)** *Pmch* level does not correlate with its respective receptor *Mchr1*. **(H)** *Hcrt* expression significantly correlates with its receptor gene *Hcrt2* in TB_high_ mice.

**Figure 7:**
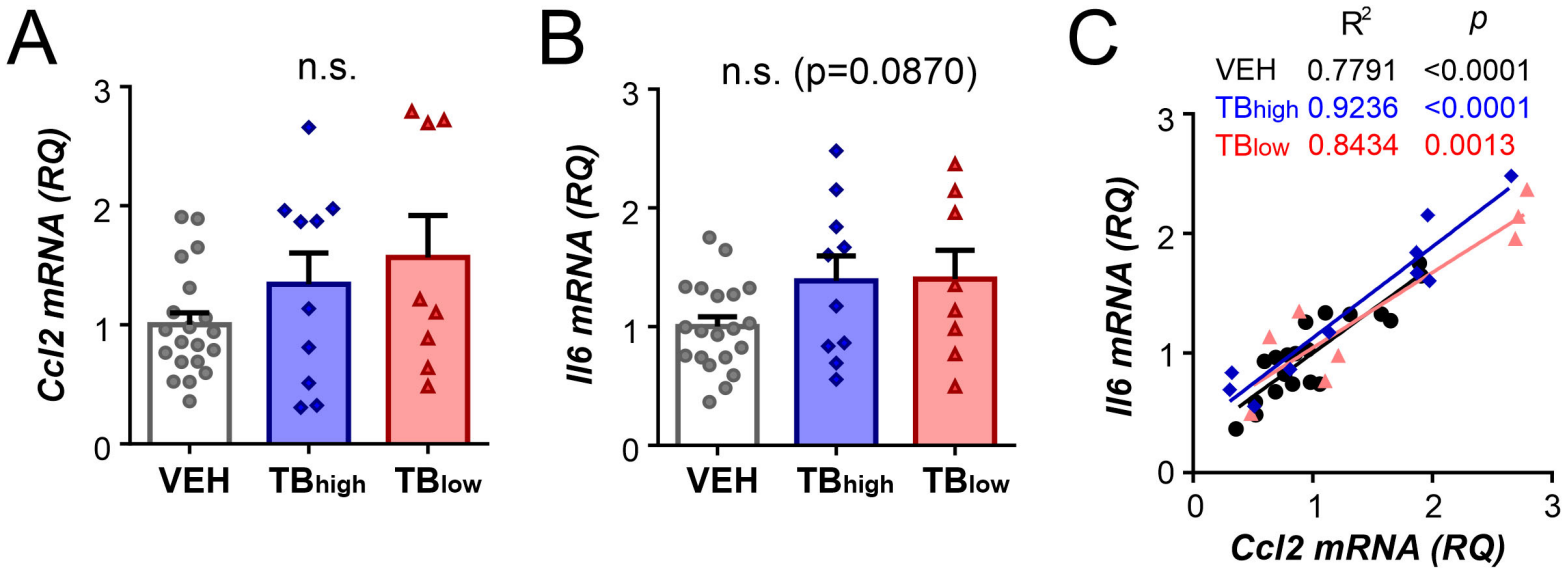
Hypothalamic expression of inflammatory cytokine genes Ccl2 and Il6 is not affected by the presence of tumor. Relative mRNA expression of *Ccl2* **(A)**, and *Il6* **(B)** in vehicle control and TB mice. Measured values were normalized to GAPDH, then normalized to respective age-matched vehicle controls. Symbols depict individual mice. Mean ± SEM are shown. n.s.: no significant main effect. **(C)** *Ccl2* expression tightly correlates with *Il6* in vehicle control and TB mice.

To further evaluate the gene expression changes, correlations between closely related genes were assessed. The peptide products of *Pomc* and *Agrp*, expressed by arcuate neurons, are known to act antagonistically on melanocortin receptors (5). In our study, these genes showed positive correlation in VEH controls, but no correlation was found in TB groups (VEH n=20, TB_high_ n=10, TB_low_ n=7, Pearson correlation VEH: p<0.0001, TB_high_: p=0.1899, TB_low_ p=0.1121; **Figure 6A**). *Pomc* was positively correlated with melanocortin receptor *Mc3r* in controls and TB_high_ (VEH: p=0.0076, TB_high_: p=0.0096, TB_low_ p=0.6941; **Figure 6B**), while not correlated with *Mc4r* in any of the groups (VEH: p=0.0466, TB_high_: p=0.6256, TB_low_ p=0.5151; **Figure 6C**). In contrast, in TB groups, *Agrp* did not correlate with either *Mc3r* (VEH: p=0.0037, TB_high_: p=0.1246, TB_low_ p=0.6005; **Figure 6D**) or *Mc4r* (VEH: p=0.0833, TB_high_: p=0.8215, TB_low_ p=0.7387; **Figure 6E**). The lateral hypothalamic genes (*Pmch* and *Hcrt*) were strongly correlated in controls and TB_high_ groups but not in TB_low_ (VEH: p=0.0002, TB_high_: p<0.0001, TB_low_ p=0.5399; **Figure 6F**). While *Pmch* expression was not correlated with MCH receptor *Mchr1* (VEH: p=0.6204, TB_high_: p=0741, TB_low_ p=0.4789; **Figure 6G**), *Hcrt* was highly correlated with hypocretin receptor *Hcrtr2* expression in control and TB_high_ groups (VEH: p=0.0042, TB_high_: p=0.0008, TB_low_ p=0.8077; **Figure 6H**). Hypothalamic inflammation can contribute to cancer-associated anorexia and cachexia in tumor-bearing mouse models. Pro-inflammatory cytokines, such as interleukin-6 (IL-6), and chemokines, such as C-C motif chemokine ligand 2 (CCL2), have been shown to disrupt hypothalamic control of energy balance (25–27). Therefore, we next assessed by RT-qPCR the mRNA levels of these two genes in the hypothalamus (**Figure 7**). The presence of tumor did not alter the expression of *Ccl2* mRNA (VEH n=20, TB_high_ n=10, TB_low_ n=8, Kruskal-Wallis test p=0.3797, **Figure 7A**). Furthermore, *Il6* mRNA level showed a trend but not a significant difference among the groups (one-way ANOVA p=0.0870, **Figure 7B**). However, *Ccl2* was highly correlated with *Il6* expression in controls and TB groups (Pearson correlation VEH: p<0.0001, TB_high_: p<0.0001, TB_low_: p=0.0013, **Figure 7C**).

**Figure 8:**
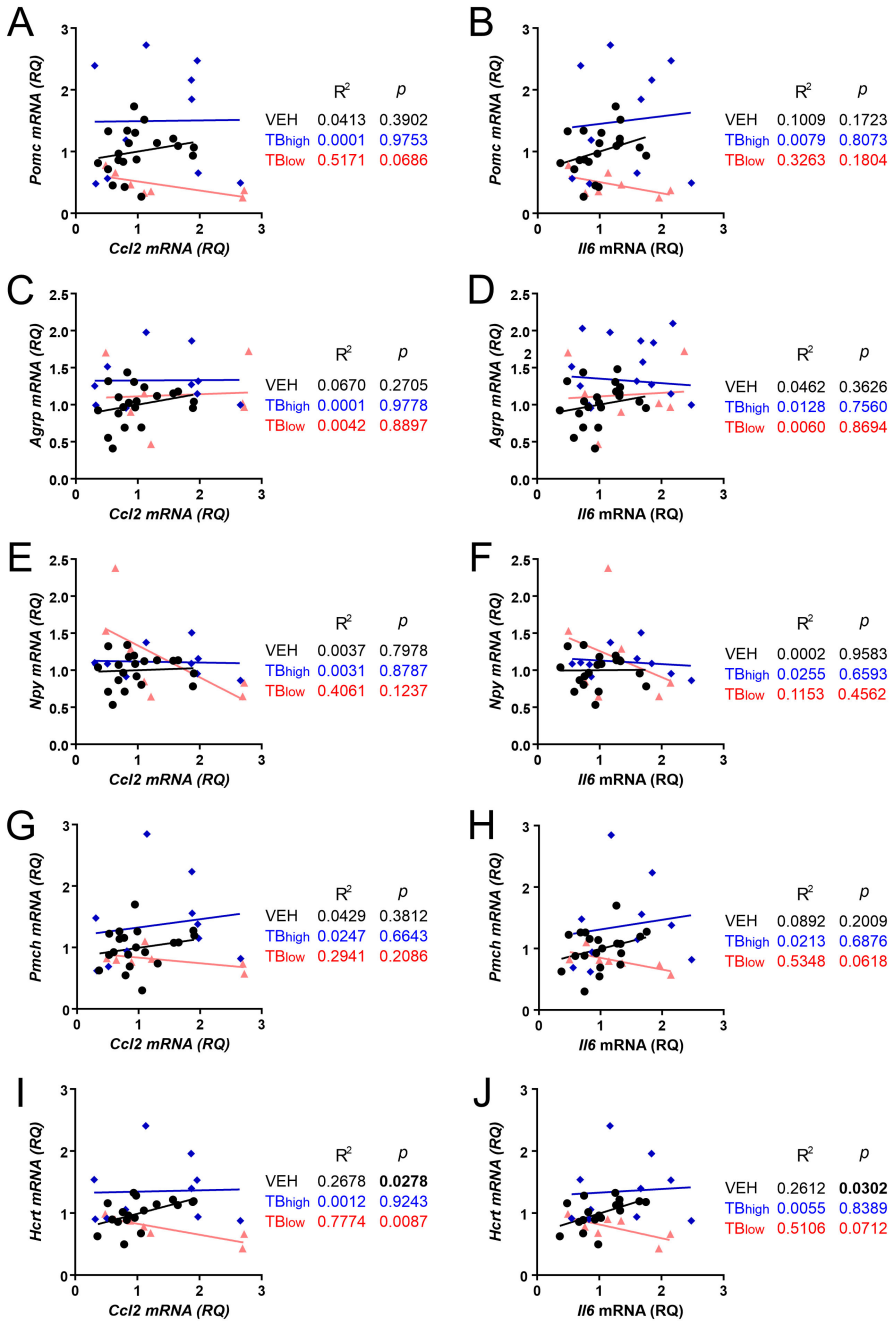
Relationship between inflammatory cytokines and feeding neuropeptide gene expression in the hypothalamus. Expression of feeding-related arcuate neuropeptide genes *Pomc*, *Agrp* and *Npy* correlate with neither hypothalamic *Ccl2* **(A, C, E)** nor *Il6* mRNA levels **(B, D, F)** in control and TB mice. Similarly, *Pmch* shows no significant correlation with *Ccl2* **(G)** or *Il6* **(H)**. *Hcrt* is negatively correlated with *Ccl2* in TB_low_ group **(I)**, but not with *Il6* **(J)**.

We further investigated whether *Ccl2* and/or *Il6* mRNA levels correlated with the expression of energy balance-related neuropeptides (**Figure 8**). None of the arcuate neuropeptide genes showed a correlation with the inflammatory genes: *Pomc* vs. *Ccl2* (VEH n=20, TB_high_ n=10, TB_low_ n=7, VEH: p=0.3902, TB_high_: p=0.9753, TB_low_: p=0.0686, **Figure 8A**), *Pomc* vs. *Il6* (VEH: p=0.1723, TB_high_: p=0.8073, TB_low_: p=0.1804, **Figure 8B**), *Agrp* vs. *Ccl2* (VEH: p=0.2705, TB_high_: p=0.9778, TB_low_: p=0.8897, **Figure 8C**), *Agrp* vs. *Il6* (VEH: p=0.3626, TB_high_: p=0.7560, TB_low_: p=0.8694, **Figure 8D**), *Npy* vs. *Ccl2* (VEH: p=0.7978, TB_high_: p=0.0887, TB_low_: p=0.1237, **Figure 8E**), *Npy* vs. *Il6* (VEH: p=0.9583, TB_high_: p=0.6593, TB_low_: p=0.4562, **Figure 8F**). Similarly, lateral hypothalamic *Pmch* correlated with neither *Ccl2* (VEH: p=0.3812, TB_high_: p=0.6643, TB_low_: p=0.2086, **Figure 8G**) nor *Il6* (VEH: p=0.2009, TB_high_: p=0.6876, TB_low_: p=0.0618, **Figure 8H**). In contrast, *Hcrt* and *Ccl2* expression showed a significant positive correlation in controls whereas negatively correlated in TB_low_ groups (VEH: p=0.0278, TB_high_: p=0.9243, TB_low_: p=0.0087, **Figure 8I**). While *Il6* was positively correlated with *Hcrt* in control group, no significant correlation was observed in TB groups (VEH: p=0.0302, TB_high_: p=0.8389, TB_low_: p=0.0712, **Figure 8J**).

In summary, *Pomc* and *Hcrt* mRNA were reduced in TB_low_ group (low initial tumor load), but these changes were not correlated with their respective receptor mRNA. In contrast, in TB_high_ group (high initial tumor load), these peptide genes and corresponding receptors did not differ from controls, whereas *Agrp* was upregulated. These changes may represent counterregulatory responses to tumor-induced energy imbalance in mice. Contrastingly, *Ccl2* and *Il6* mRNA levels were not significantly different among the groups, although their expression was highly correlated to each other in all groups. Furthermore, no correlation was observed for either *Ccl2* or *Il6* with most feeding peptide genes tested. One exception was *Hcrt*, which was negatively correlated with *Ccl2* in TB_low_ group, suggesting a possible role of CCL2 in the anorexic phenotype of this mouse model.

## 4 Discussion

The present study shows that the initial tumor load influences the metabolic phenotype and hypothalamic response. Major differences between high and low tumor load were the time to reach the endpoint and the magnitude of weight loss and anorexia. Not surprisingly, TB_high_ group reached the endpoint faster than TB_low_ group. Total body weight change was positively correlated with food intake in TB_high_ mice, but not in TB_low_ mice. On the other hand, both TB groups displayed significantly lower food efficiency compared to controls, which was tightly correlated with lower body weight. These results suggest that the primary reason for diminished weight gain in LLC-TB mice is elevated energy expenditure. While food efficiency is not a direct measure of energy expenditure and does not necessarily reflect altered metabolism, our results are consistent with a previous report demonstrating adipose tissue browning and enhanced energy expenditure in LLC-TB model (20). As the only difference between the two TB groups is the number of implanted cancer cells, subsequent divergence in tumor growth rate, tumor exposure duration and host response can be attributed to the initial tumor load.

In previous reports that used 1 x 10^6^ LLC cells (equivalent to our TB_high_ model), the time to display cachexia ranged from 2–3 weeks (15, 16) to 4 weeks post-tumor implantation (18, 19, 23, 24). These differences may be due to age, as middle-aged mice (15, 16) displayed cachexia and reached the end point earlier than young adult mice (23, 24). However, our study tested young adult mice (8-week-old) that showed a decrease in body weight gain and some organ mass within 2 weeks, suggesting that other factors may also influence the time course of the metabolic phenotype of this mouse model. It may be relevant that LLC cells originated from a male C57BL/6L mouse (a transitional strain between C57BL/6J and C57BL/6N) (28), and aforementioned previous studies utilized C57BL/6J strain or transgenic mice on a C57BL/6 background, whereas C57BL/6NCrl strain was used in our study.

Using these LLC-TB models, we investigated the expression of neuropeptide and receptor genes, and inflammatory mediator genes in the hypothalamus that are known to be sensitive to homeostatic challenges. In TB_high_ mice, *Agrp* mRNA expression was upregulated compared to VEH controls, which may be a compensatory response to resist weight loss, similar to a reported study on a rat cancer model (29). Other neuropeptide genes in this group did not significantly differ from those in controls but showed a high individual variability for *Pomc* and *Hcrt*, which was positively correlated with their corresponding receptors.

In contrast to TB_high_ mice, TB_low_ mice displayed a significant reduction in *Pomc* and *Hcrt* mRNA. *Hcrt* downregulation is consistent with a previous microarray analysis study on a LLC-TB mouse model equivalent to our TB_low_ mice, although their study found no change in *Pomc* expression (22). As both hypocretin and the main POMC product α-melanocyte-stimulating hormone (α-MSH) promote lower body weight (30, 31), their downregulation may potentially reflect a counterregulatory response to limit weight loss. On the other hand, we found no change in orexigenic *Agrp*, *Npy* or *Pmch* mRNA expression in this group, suggesting that the hypothalamus may not be actively promoting food intake and weight gain. Taken together, these results suggest a homeostatic role for these neuropeptides to counter a weight loss due to a tumor.

Inflammation is one of the primary factors driving brain changes in the presence of non-brain malignancy (30). Peripheral inflammation may lead to acute and chronic inflammation in the brain, particularly the hypothalamus, which may in turn act to maintain energy homeostasis or induce sickness responses (31). Circulating proinflammatory cytokines, such as tumor necrosis factor-α and IL-6, are known to be upregulated in LLC-TB mouse model (21). It has been previously shown that these cytokines can influence orexigenic and anorexigenic hypothalamic neuropeptides, including NPY, AgRP, and POMC, thereby suppressing appetite and contributing to anorexia and cachexia (32–34). Therefore, hypothalamic levels of *Ccl2* and *Il6* mRNA were investigated, which showed no significant differences in their expression levels among the groups. Nevertheless, in TB_low_ mice, *Ccl2* expression displayed a significant negative correlation with *Hcrt* and a tendency to correlate with *Pomc* (p=0.0686). Other neuropeptide genes did not show any correlation with *Ccl2* or *Il6* mRNA expression in either TB group. Overall, the influence of these inflammatory mediators on neuropeptide mRNA expression appears to be minimal in TB_high_ mice, while CCL2 may play a modest role in inhibiting *Hcrt* and *Pomc* expression in TB_low_ mice. Indeed, previous studies have suggested that the influence of IL-6 and CCL2 on hypothalamic gene expression may vary by tumor type and be model-dependent (35). It is possible that alternative inflammatory mediators, including IL-1β, TNF-α, or GDF15, predominate in driving anorexic signaling in LLC-TB mice (36, 37).

Another potential change that may mediate the effect of tumor on the hypothalamus is reduced adiposity resulting in lower levels of circulating leptin. This may impact leptin-sensitive hypothalamic neurons, particularly AgRP and POMC neurons in the arcuate nucleus and their downstream signaling (38, 39). It is important to note that mRNA expression levels do not always correlate with protein abundance or functional alteration of a cell. Therefore, future studies should assess changes in the expression levels of proteins involved in energy balance regulation and inflammation under different tumor burden.

In conclusion, our findings provide insight into coordinated response of energy balance-related hypothalamic neurons under TB conditions. In LLC-TB mice, the hypothalamus may elicit counterregulatory responses, which could be influenced by dynamic changes in energy substrate availability, inflammation, and hormonal landscape. By making a direct comparison of LLC-TB mouse models with different numbers of implanted cancer cells, our study demonstrates that a greater initial tumor load does not necessarily result in more severe metabolic phenotype or changes in the brain. Thus, while alterations in systemic energy metabolism tend to become more pronounced with increasing tumor burden and advancing disease, the relationship between energy balance and cancer progression is complex and not strictly linear. This finding should be carefully considered in the design and interpretation of future animal experiments and clinical studies.

## Data Availability

The original contributions presented in the study are included in the article/**Supplementary Material**. Further inquiries can be directed to the corresponding author.
