# Supplementary material for: Dependence of energy balance and hypothalamic neuropeptide gene expression on initial tumor load in mice
Source: Front Oncol. 2026 May 19;16:1783555. doi: 10.3389/fonc.2026.1783555 (PMC13226006; doi:10.3389/fonc.2026.1783555)
Supplement: Supplementary file 1 [file DataSheet1.docx]

Supplementary Material

**
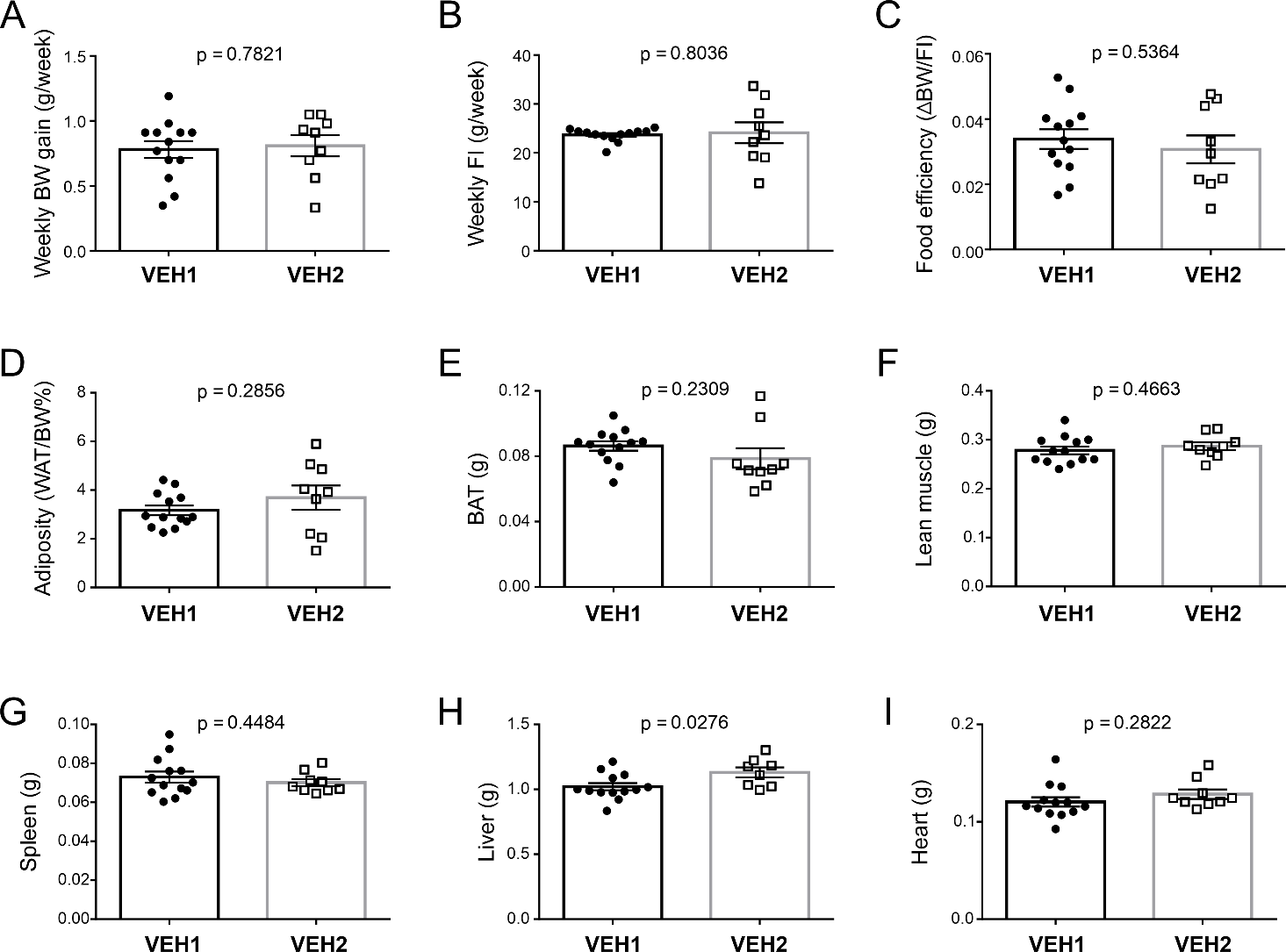
**

**Supplementary Figure 1.** **Comparison of vehicle control mice.**

Control mice, age-matched to TB_high_ (VEH1) or TB_low_ (VEH2) are compared. A-D) Weekly BW gain (A), food intake (FI, B) food efficiency (C) and adiposity (D) are not different. E-I) Weight of the brown adipose tissue (BAT) (E), lean muscles (F), liver (G), heart (H), and spleen (I). Organ weights are not different between the groups except for the liver mass. Each symbol represents individual mouse. Mean ± SEM are shown.
